# Identification of Potential Hub Genes Related to Diagnosis and Prognosis of Hepatitis B Virus-Related Hepatocellular Carcinoma via Integrated Bioinformatics Analysis

**DOI:** 10.1155/2020/4251761

**Published:** 2020-12-08

**Authors:** Yuqin Tang, Yongqiang Zhang, Xun Hu

**Affiliations:** ^1^School of Basic Medical Sciences, Chengdu University of Traditional Chinese Medicine, Chengdu 611137, China; ^2^Biorepository, State Key Laboratory of Biotherapy and Cancer Center, West China Hospital, Sichuan University, Chengdu 610041, China; ^3^Molecular Medicine Center, West China Hospital, Sichuan University, Chengdu 610041, China; ^4^West China School of Medicine, West China Hospital, Sichuan University, Chengdu 610041, China

## Abstract

Hepatocellular carcinoma (HCC) is a common malignant cancer with poor survival outcomes, and hepatitis B virus (HBV) infection is most likely to contribute to HCC. But the molecular mechanism remains obscure. Our study intended to identify the candidate potential hub genes associated with the carcinogenesis of HBV-related HCC (HBV-HCC), which may be helpful in developing novel tumor biomarkers for potential targeted therapies. Four transcriptome datasets (GSE84402, GSE25097, GSE94660, and GSE121248) were used to screen the 309 overlapping differentially expressed genes (DEGs), including 100 upregulated genes and 209 downregulated genes. Gene Ontology (GO) and Kyoto Encyclopedia of Genes and Genomes (KEGG) enrichment were used to explore the biological function of DEGs. A PPI network based on the STRING database was constructed and visualized by the Cytoscape software, consisting of 209 nodes and 1676 edges. Then, we recognized 17 hub genes by CytoHubba plugin, which were further validated on additional three datasets (GSE14520, TCGA-LIHC, and ICGC-LIRI-JP). The diagnostic effectiveness of hub genes was assessed with receiver operating characteristic (ROC) analysis, and all hub genes displayed good performance in discriminating TNM stage I patient samples and normal tissue ones. For prognostic analysis, two prognostic key genes (TOP2A and KIF11) out of the 17 hub genes were screened and used to develop a prognostic signature, which showed good potential for overall survival (OS) stratification of HBV-HCC patients. Gene Set Enrichment Analysis (GSEA) was performed in order to better understand the function of this prognostic gene signature. Finally, the miRNA–mRNA regulatory relationships of all hub genes in human liver were predicted using miRNet. In conclusion, the current study gives further insight on the pathogenesis and carcinogenesis of HBV-HCC, and the identified DEGs provide a promising direction for improving the diagnostic, prognostic, and therapeutic outcomes of HBV-HCC.

## 1. Introduction

Liver cancer, with about 841,000 new cases diagnosed and 782,000 deaths in 2018, still represents a common lethal solid tumor and ranks fourth leading cause of cancer-related deaths worldwide [1]. In China, liver cancer was one of the first five life killers in 2017 [2]. Hepatocellular carcinoma (HCC), comprising 75%-85% of all primary liver cancer cases worldwide [1], is the primary histological subtype. The major causative etiological factors of HCC are considered as infection of endemic hepatitis B virus (HBV) or hepatitis C virus (HCV), followed by exposition to aflatoxin B1, alcohol abuse, and obesity [1, 3]. Particularly, HBV infection is considered the dominant cause of HCC, accounting for more than 80% of all HCC incidences in China and other developing countries [4]. Despite significant advances in early diagnosis, prevention and the standard therapeutic interventions such as surgery, radiation, chemotherapy, or personalized target therapeutic strategies developed during the last decade, and the cumulative 5-year overall survival rate of HCC remains unfavorable, probably due to its invasive behavior, as well as its histopathological and molecular heterogeneity that challenge molecular characterization and targeted therapeutic approaches. Moreover, the majority of patients are diagnosed in a more advanced stage, resulted in a much poorer prognosis. Thus, considerable work is still required to achieve a better understanding of the underlying mechanism at molecular level on the pathogenesis and carcinogenesis of HCC, which may be imperative for the development of robust biomarkers for early diagnosis and drug discovery.

In recent years, the rapid development of bioinformatics and emerging high-throughput techniques, such as microarray and next-generation sequencing (NGS), have enabled us to gain a comprehensive understanding of carcinogenesis and progression of various types of cancer. High-throughput platforms have been widely used in early diagnosis, histological identification, molecular classification, prognosis prediction, and drug resistance analysis of cancer [5–9]. Differentially expressed genes (DEGs), microRNAs (miRNAs), long noncoding RNAs (lncRNAs), and circular RNAs (circRNAs), as well as differentially methylated CpG sites have the potential to provide valuable clues to locate biomarkers in HCC. Nevertheless, data acquired from multiple studies may lead to false-positive results, considering the sample heterogeneity, different screening methods, different data mining approaches, and coupled effect of limited sample size in a single independent study. Integrated analysis based on collective datasets is a promising strategy to overcome these shortcomings. The microarray and sequencing data that deposited in the public databases such as The Cancer Genome Atlas (TCGA), Gene Expression Omnibus (GEO), and International Cancer Genome Consortium (ICGC) have generated valuable information to identify biomarkers or to explore molecular landscapes of cancers, especially at transcriptome level [10–15]. Integrated transcriptome analyses have been employed to disclose the molecular mechanisms of cancers. However, few reports have been submitted to examine dysregulated genes and candidate biomarkers regarding HBV-related HCC (HBV-HCC) with combined datasets [16].

In the current study, seven datasets of gene expression profiles from GEO, TCGA, and ICGC were used to identify the hub genes that may play pivotal roles in the pathogenesis of HBV-HCC, comprising a total of 514 HBV-HCC tumor samples and 452 normal tissues. The common differentially expressed genes between tumors and adjacent normal liver tissues of four datasets (GSE84402 [17], GSE25097 [18], GSE94660 [19], and GSE121248 [20]) were screened by the R language software. The R package clusterProfiler [21] was utilized to conduct Gene ontology (GO) enrichment analysis and the Kyoto Encyclopedia of Genes and Genomes (KEGG) pathway analysis to reveal the biological functions of DEGs. The protein-protein interaction (PPI) network of all common DEGs was constructed with information from the Search Tool for the Retrieval of Interacting Genes (STRING) database. Based on the entire network, hub gene identification and submodule analysis were performed using the CytoHubba plugin or the Molecular Complex Detection (MCODE) plugin of Cytoscape [22–24], respectively. The expression levels and correlation analysis of hub genes were then validated with the aid of complementary three datasets (TCGA, ICGC, and GSE14520). Furthermore, the diagnostic effectiveness of the hub genes was evaluated from TCGA and GSE14520, and the prognostic value was also determined in the GSE14520 dataset using univariate and multivariate analyses. Finally, Gene Set Enrichment Analysis (GSEA) and mRNA–microRNA interactions were operated to further clarify their possible molecular mechanisms. Taken all together, the crucial hub genes and associated key pathways identified from our findings would provide a potential indication for the molecular mechanism of HBV-HCC development and progression, and the particular proposed genes may shed additional insight into the early diagnosis, prognosis prediction, and therapeutic targets of HBV-HCC in the near future.

## 2. Materials and Methods

### 2.1. Gene Expression Dataset Acquisition

We chose four datasets of gene expression profiling from the GEO (https://www.ncbi.nlm.nih.gov/geo/) database to fetch the DEGs between HBV-HCC tumor samples and normal liver tissues, with the accession numbers of GSE84402, GSE25097, GSE94660, and GSE121248. GSE84402 and GSE121248 were both based on GPL570 (Affymetrix Human Genome U133 Plus 2.0 Array), while GSE25097 was based on GPL10687 (Merck Human RSTA Affymetrix 1.0 microarray, Custom CDF), and GSE94660 was based on GPL16791 (Illumina Hiseq 2500 (Homo sapiens)). HBV-HCC cases or samples were carefully filtered from all of the above studies. The dataset of GSE84402 contained 13 pairs of HBV-related hepatocellular carcinoma tissues and corresponding noncancerous tissues. The dataset of GSE25097 comprised 73 HBV-HCC tissues and 67 paired adjacent nontumor samples. The dataset of GSE94660 consisted of 21 pairs of tumor and nonneoplastic liver tissues of HBV-HCC patients. The dataset of GSE121248 included 70 tissues from chronic hepatitis B-induced HCC and 37 adjacent normal tissues. In addition, we downloaded another three transcriptome datasets and corresponding clinical information for validation—GSE14520 from the GEO database including 213 HBV-HCC tissues and 220 normal samples, TCGA-LIHC data from The Cancer Genome Atlas (TCGA, http://www.tcga.org/) database including 70 HBV-HCC tissues and 49 normal samples, and ICGC-LIRI-JP data from the International Cancer Genome Consortium (ICGC, https://icgc.org/) including 53 HBV-HCC tissues and 45 paired normal samples.  Table 1 summarized the detailed information of the selected datasets in this study.

### 2.2. Data Preprocessing and DEG Identification

All data were analyzed by the R software (version3.6.0, https://http://www.r-project.org/). For the microarray data, we downloaded the raw data (.CELL files) and used the robust multiarray average algorithm [25] to conduct the background correction and quantile normalization; probes were subsequently matched with the corresponding gene name according to the platform annotation packages. When multiple probes were matched one the same gene, the highest expression value of all probes was selected to represent the final expression level. For RNA-seq data, we collected the normalized gene expression matrixes from the GEO database or HCCD database [26] directly. Meanwhile, the limma package [27] was applied to screen DEGs between HBV-HCC samples and noncancerous samples, with *P* value <0.05 and ∣log2 FC | ≥1 were set as the statistical cutoff criteria. The overlapping DEGs were identified by Venn plot. Besides, to minimize the chance for making type I error, we also adopted the integrating strategy by removing batch effect via “sva” [28] package for the three microarray datasets.

### 2.3. Functional Enrichment Analysis

To explore a further understanding of the potential biological function, GO and KEGG pathway enrichment analyses were performed on the overlapping dysregulated genes using the package “clusterProfiler” [21], and *P* < 0.05 was determined as a cutoff for significance.

### 2.4. PPI Network Construction

STRING database (version 11.0; http://string-db.org/), a free online tool to evaluate PPI information, was used to assess and integrate the interactive relationships among these DEGs. A combined confidence score of ≥0.7 (high confidence) was set as the threshold value. The Cytoscape software [24] (version 3.2.1; http://www.cytoscape.org) was then used to construct and visualize the PPI network. Besides, we also used a plugin from Cytoscape, the MCODE [23] (MCODE; version 1.5.1), to carry out the submodule analysis. For the identification of hub genes, we used the CytoHubba app [22] in Cytoscape with a combined method. Generally, nodes with top 60 scores of all the 11 algorithms in CytoHubba were picked up; then, the intersection based on more than 8 algorithms was taken as candidate hub genes, and two packages including UpSetR [29] and Venn detail [30] were used for visualization. Finally, the potential function of submodule genes or the hub genes was verified and displayed by the clusterProfiler [21] and the GOplot R packages [31].

### 2.5. Validation of Hub Gene Expression

As mentioned above, three datasets including GSE14520, TCGA-LIHC, and ICGC-LIRI-JP were used to verify the differential expression level of hub genes. The violin plots and heatmaps were drawn to compare the expression pattern between HBV-HCC tissues and noncancerous tissues, with the Wilcoxon test to measure the statistical significance. The same three datasets were also used to perform the coexpression analysis of the selected hub genes. The correlation of hub genes' expression and the clinical stages was investigated with the GSE14520 dataset and exhibited with boxplots.

### 2.6. Assessment of the Diagnostic and Prognostic Values

The receiver operating characteristic (ROC) analysis was conducted by means of R package pROC [32] to determine the power of potential hub genes in the diagnose of early phrase and whole phrase of HBV-HCC carcinogenesis, using their expression values from GSE14520 and TCGA-LIHC. The appropriate expression levels of the hub genes were served as cutoff values. For prognostic assessment, because of the sample size (*n* < 100) limitation of other datasets, only GSE14520 containing 213 HBV-HCC patients with complete OS data and sufficient clinicopathologic information were analyzed. In order to increase the robustness of the selection, we applied the “multi-split” strategy with log-rank test for 100 randomizations (75% portion of all samples were subsampled at each time) to evaluate the correlation between the OS and each hub gene expression level (Supplementary Figure 1). Those genes repeatedly showed significance for more than 75 times were considered as prognostic key genes and were further used to construct a linear combination as the risk signature with the following formula: risk score = sum (coef (*k*) × expression value of *k*), where *k* represents the candidate prognostic key genes. All patients were divided as high-risk or low-risk groups according to the median risk score. Kaplan-Meier method with a log-rank test was used to compare the survival curves by using the survival package [33]. The time-dependent ROC was depicted to estimate the predictive ability of the risk signature for patients' OS survival. The associations of clinicopathologic features and the risk signature were determined by Pearson chi-square test or Fisher's exact test. Univariable and multivariable Cox regression analyses were conducted to identify independent prognostic factors. For all statistical tests, *P* < 0.05 was set as significant cutoff.

### 2.7. Gene Set Enrichment Analysis

The genes that differentially expressed and associated with risk stratification of OS survival were analyzed by GSEA, which was employed to examine the statistical significance of a priori defined set of genes between different phenotypes. In the current study, we focused on the KEGG pathways (c2.cp.kegg. v7.1) and molecular function of GO gene sets (c5.mf.v7.1). 1000 times of permutations were performed to obtain the enrichment score (ES) and the normalization enrichment score (NES) for each gene set. Significant gene sets were identified with a nominal *P* value <0.05, combined with an FDR < 25% and normalized enrichment score (NES) > 1 or <-1.

### 2.8. miRNA-mRNA Interaction Prediction

We predicted the liver-specific miRNA-mRNA interaction of all hub genes using an online tool—miRNet [34], which integrated several well-annotated databases, including miRTarBase v7.0, TarBase v7.0, and miRecords. miRNA-mRNA correlation network was constructed with the Cytoscape software. In the network, diamond nodes denoted the mRNAs while the rectangle nodes represented the miRNAs.

## 3. Results

### 3.1. Identification of DEGs in HBV-HCC

**Figure 1:**
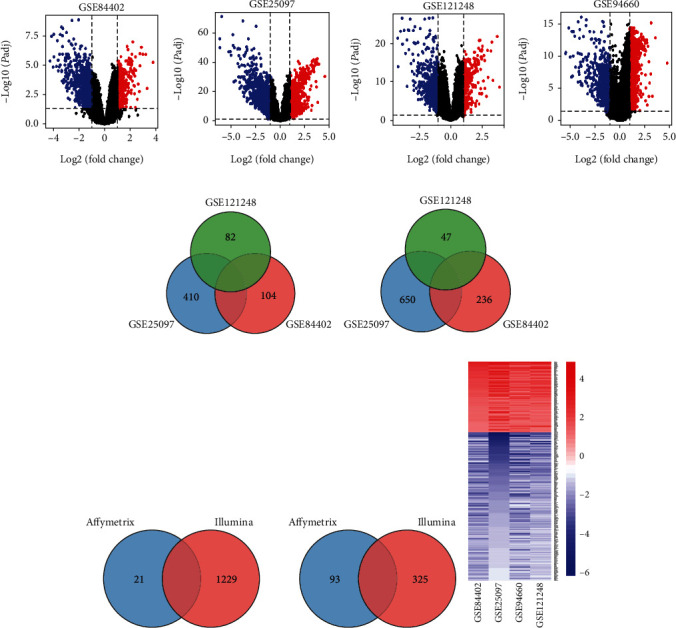
Identification of overlapping DEGs among the screening datasets of HBV-HCC. (a–d) Volcano plot for GSE84402, GSE25097, GSE121248, and GSE94660. (e) Venn diagram for the overlapping DEGs from Affymetrix biosystems. (f) Common DEGs shared by Affymetrix and Illumina platforms. (g) Heatmap of 309 common DEGs. Blue represents downregulated genes while red represents upregulated genes. Each column represents one dataset, and each row represents one gene. DEGs: differentially expressed genes; HBV-HCC: HBV-related hepatocellular cancer.

With the filtering criteria mentioned above, DEGs in HBV-HCC carcinogenesis were achieved using a total of 315 clinical samples from four GEO datasets (GSE25097, GSE84402, GSE121248, and GSE94660). In all, there were 623 upregulated and 1144 downregulated genes identified from GSE25097, 319 upregulated and 775 downregulated genes from GSE84402, 319 upregulated and 564 downregulated genes from GSE121248, and 1329 upregulated and 534 downregulated genes from GSE94660. The volcano plots of all DEGs in each of the four datasets were shown in Figures 1(a)–1(d). For overlapping analysis of the Venn diagram, 121 upregulated genes or 302 downregulated genes were firstly shared by Affymetrix biosystems (Figure 1(e)); then, 309 common DEGs were obtained by Affymetrix and Illumina platforms, consisting of 100 upregulated genes and 209 downregulated genes (Figures 1(f) and 1(g)). To increase the robustness of these 309 DEGs, we conducted the integrating analysis of the three microarray datasets. After removing the batch effect, all 309 DEGs were still showed significant (Supplementary Figure 2, Supplementary Table 1).

### 3.2. DEG Function Analysis

**Figure 2:**
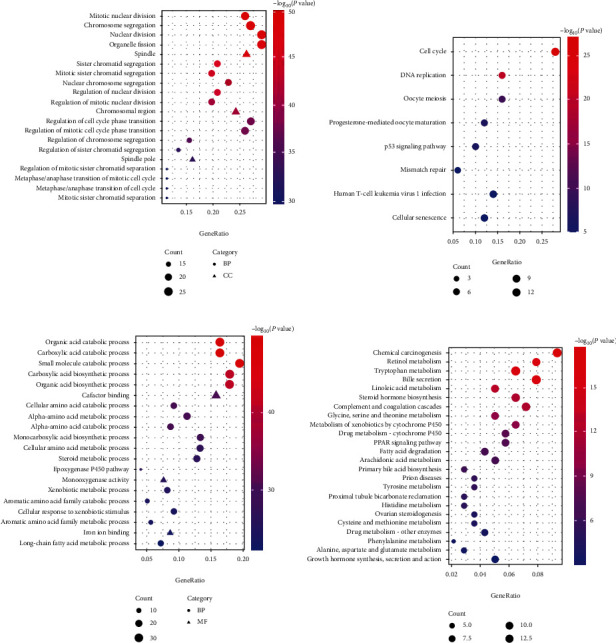
Function enrichment analysis of the overlapping DEGs. (a) GO analysis for the upregulated DEGs (top 20 GO terms are shown). (b) KEGG pathway enrichment for the upregulated DEGs. (c) GO analysis for the downregulated DEGs (top 20 GO terms are shown). (d) KEGG pathway enrichment for the downregulated DEGs. BP: biological process; CC: cellular component; MF: molecular function.

Three categories of GO comprising of biological process (BP), molecular function (MF), and cellular component (CC), together with KEGG enrichment, were performed for the upregulated and downregulated genes. For GO functions analysis, upregulated genes were involved in multiple GO terms, such as mitotic nuclear division, chromosome segregation, nuclear division, and spindle. On the other hand, significant GO terms that associated with downregulated genes were organic acid catabolic process, carboxylic acid catabolic process, small molecule catabolic process, carboxylic acid biosynthetic process, and so on. For KEGG pathway analysis, the upregulated genes were mostly enriched in the cell cycle, DNA replication, oocyte meiosis, progesterone-mediated oocyte maturation, p53 signaling pathway, and mismatch repair. Meanwhile, the downregulated genes were mainly related to chemical carcinogenesis, retinol metabolism, tryptophan metabolism, bile secretion, and linoleic acid metabolism (Figure 2).

### 3.3. PPI Network Construction

**Figure 3:**
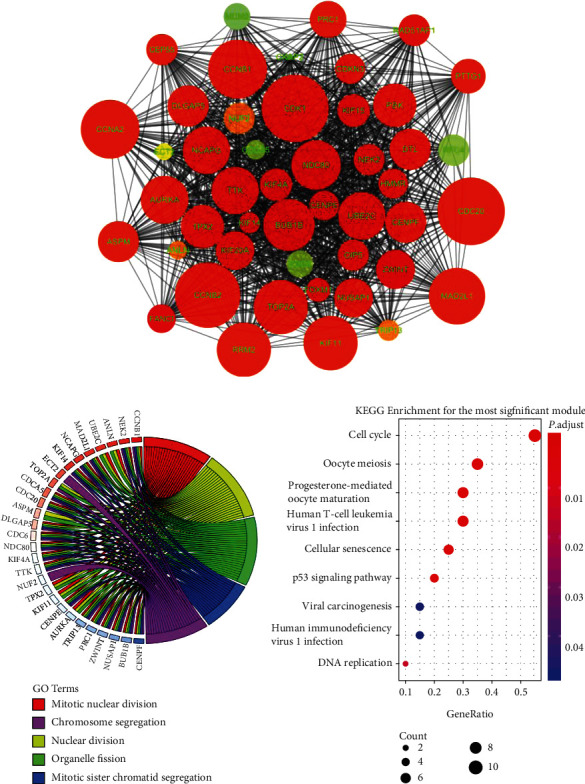
Subnetwork analysis of the DEGs PPI. (a) The most significant module selected by MCODE plugin (MCODE score > 40), comprising 47 nodes. (b) Top five related GO terms enriched by the DEGs in the module. (c) KEGG enrichment analyses for the DEGs in the module.

For the DEG interaction inspection, we constructed a PPI network of 209 nodes and 1676 edges by the STRING database and Cytoscape software, with a strict criterion (combined interaction score ≥ 0.7). The PPI network contained 84 upregulated DEGs and 125 downregulated DEGs (Supplementary Figure 3). The clustering coefficient was 0.525, and the average degree was 16.038. In addition, the MCODE app of Cytoscape detected the most significant module with a high network score (>40), which consisted of 47 nodes and 1017 edges (Figure 3(a)). Functional analysis revealed that mitotic nuclear division and cell cycle were the most significant GO and KEGG pathway enriched by the module (Figures 3(b) and 3(c)).

**Figure 4:**
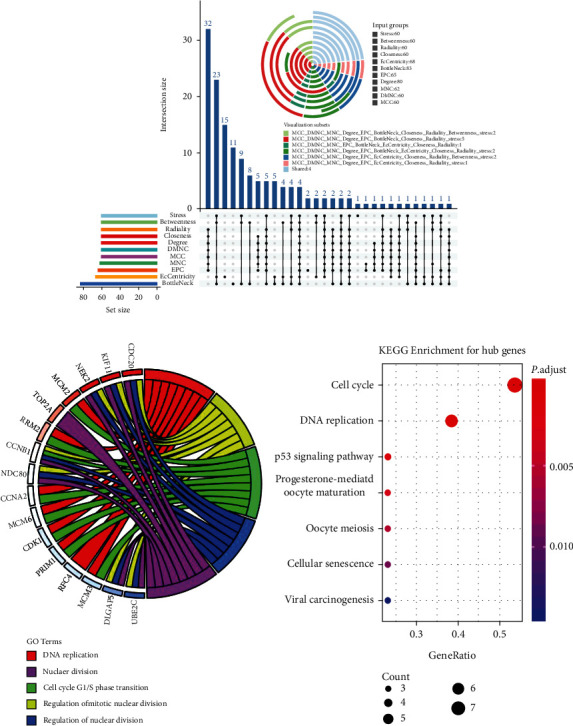
Hub gene identification and functional analysis. (a) The combination of Upset plot and Vennpie plot shows the 17 hub genes identified by CytoHubba plugin through DEG PPI network, with an overlapping strategy. (b) Top five related GO terms of the hub genes. (c) Result of KEGG pathway analysis of the hub genes.

For the hub gene identification, we used a combined method by CytoHubba. Interestingly, all of the 17 hub genes were upregulated DEGs, and most of them (UBE2C, RRM2, RFC4, TOP2A, MCM2, CDK1, CCNB1, HMMR, CDC20, CCNA2, NEK2, NDC80, DLGAP5, and KIF11) were involved in the significant module, while only 3 hub genes (MCM6, MCM3, and PRIM1) were not included (Figure 4(a)). We also employed the GO and KEGG analyses of all the hub genes for further validation of their biological functions, such as DNA replication and cell cycle regulators (Figures 4(b) and 4(c)).

### 3.4. Hub Gene Validation

**Figure 5:**
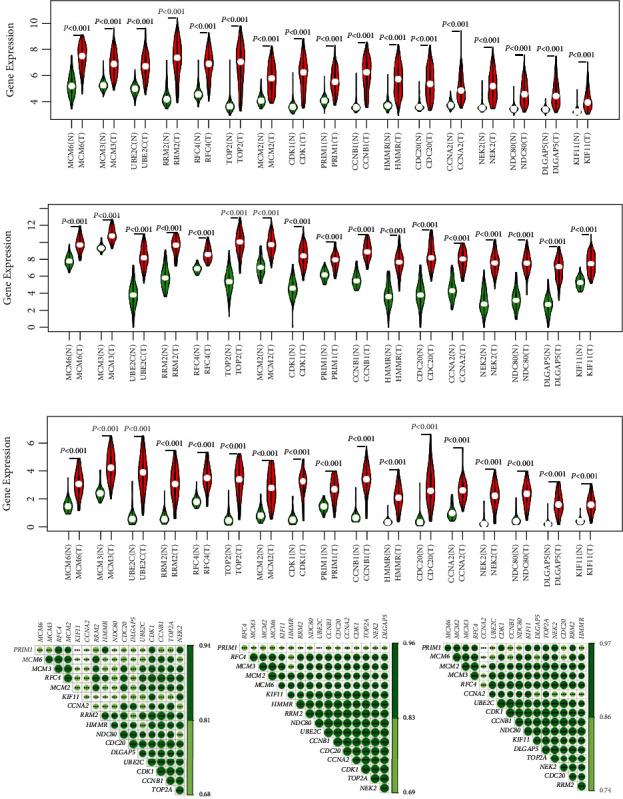
Validation of the aberrant expression levels for the selected hub genes and coexpression analysis. (a–c) Violin plots showing the significantly increased expression values for all of the 17 hub genes based on GSE14520, TCGA-LIHC, and ICGC-LIRI-JP. (d) Pearson correlation analysis among expression levels of the 17 hub genes for GSE14520, TCGA-LIHC, and ICGC-LIRI-JP. The color depth indicates the degree of correlation. The darker the color, the higher the correlation coefficient. ^∗∗∗^*P* < 0.001.

**Figure 6:**
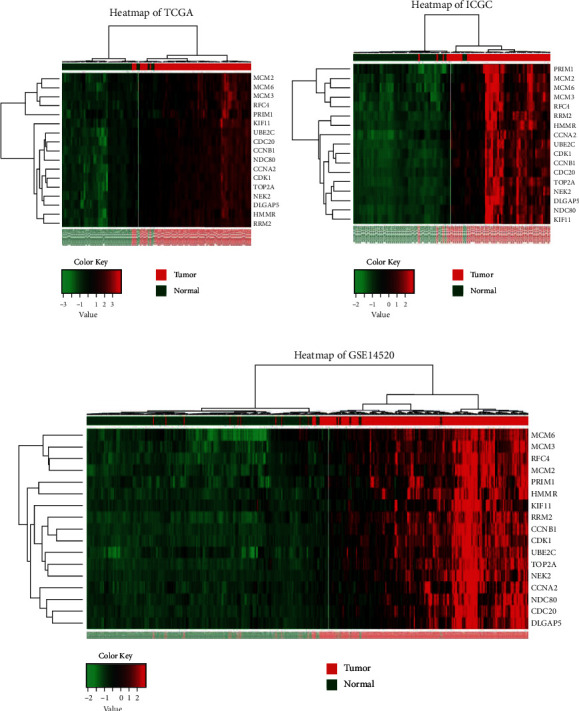
Clustering heatmaps of 17 hub genes based on (a) TCGA-LIHC, (b) ICGC-LIRI-JP, and (c) GSE14520. Red denotes high expression levels while green denotes low expression levels.

**Figure 7:**
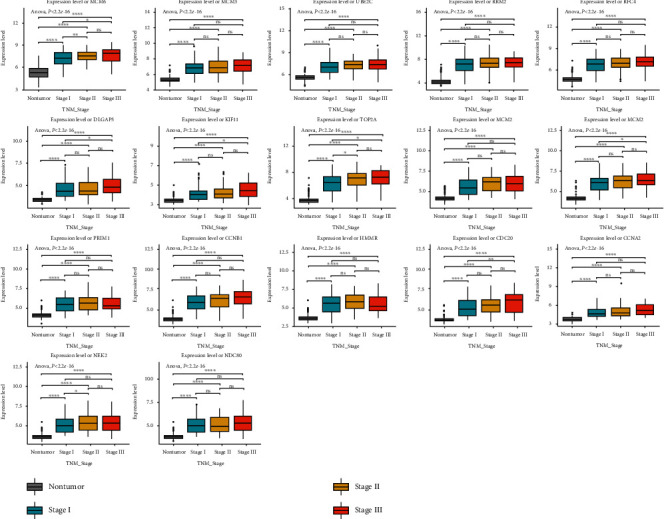
Boxplots showing the relative expression levels of 17 hub genes across normal liver tissues and cancer tissues with different TNM stages for HBV-HCC. ^∗^*P* < 0.05, ^∗∗^*P* < 0.01, ^∗∗∗^*P* < 0.001, and ^∗∗∗∗^*P* < 0.0001.

To validate the differentially expressed levels of the selected hub genes, three other datasets were used in this study. Consistent with the screening cohorts from the GEO database, all of the 17 definitive hub genes showed the significantly higher expression trends between HBV-HCC cancer samples and adjacent liver normal ones (Figures 5(a)–5(c)). Moreover, the hierarchical clustering of the hub genes and samples based on the three datasets revealed their good potential in discriminating the tumors from healthy tissues (Figure 6). Heatmaps of Pearson correlation suggested the high correlations of these hub genes in all three datasets, supporting the underlying hypothesis that hub genes may strongly interact with each other and play critical roles in the development of HBV-HCC (Figure 5(d)). To seek for the clinical relevance of these hub genes, we analyzed the transcription expression levels of the hub genes according to patients' TNM stages and their BCLC stages. Consequently, most of the hub genes showed no statistical difference between TNM stage I and other stages, which suggested their predominant roles in the initiation of carcinogenesis, but they may not good indicators for HBV-HCC progression (Figure 7). A similar result was reached based on the BCLC staging system, which implied that only MCM6, RRM2, and CCNB1 might be associated with HBV-HCV progression (Supplementary Figure 4).

### 3.5. Diagnostic Value Assessment

**Figure 8:**
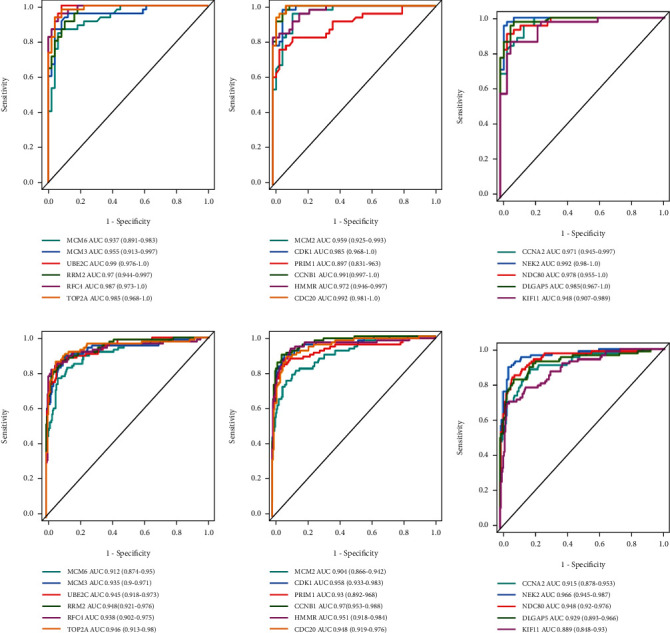
The ROC curves and AUC (95% CI) for each of the selected hub genes to evaluate their efficiency in the early diagnosis of HBV-HCC based on (a–c) TCGA-LIHC cohort and (d–f) GSE14520 cohort. Colored lines denote sensitive curves for each hub gene, and grey line denotes the identify line. ROC: receiver operating characteristic; AUC: area under the curve.

The above findings prompted us to speculate that these hub genes may have good diagnostic efficiency for HBV-HCC, which was verified by plotting ROC curves using TCGA-LIHC and GSE14520. As the result, the area under the curves (AUCs) of all hub genes ranged from 0.91 to 0.99 (Supplementary Figure 5), indicating their excellent diagnostic values for distinguishing tumor tissues and adjacent normal ones. Next, we were curious whether these hub genes also play a role in the early detection of HBV-HCC, which was even more crucial for clinical intervention. Thus, we especially focused on early stage (TNM stage I) cases, and ROC analysis of individual hub genes proved their great potential in the early diagnosis of HBV-HCC (Figure 8).

### 3.6. Survival Analysis

**Figure 9:**
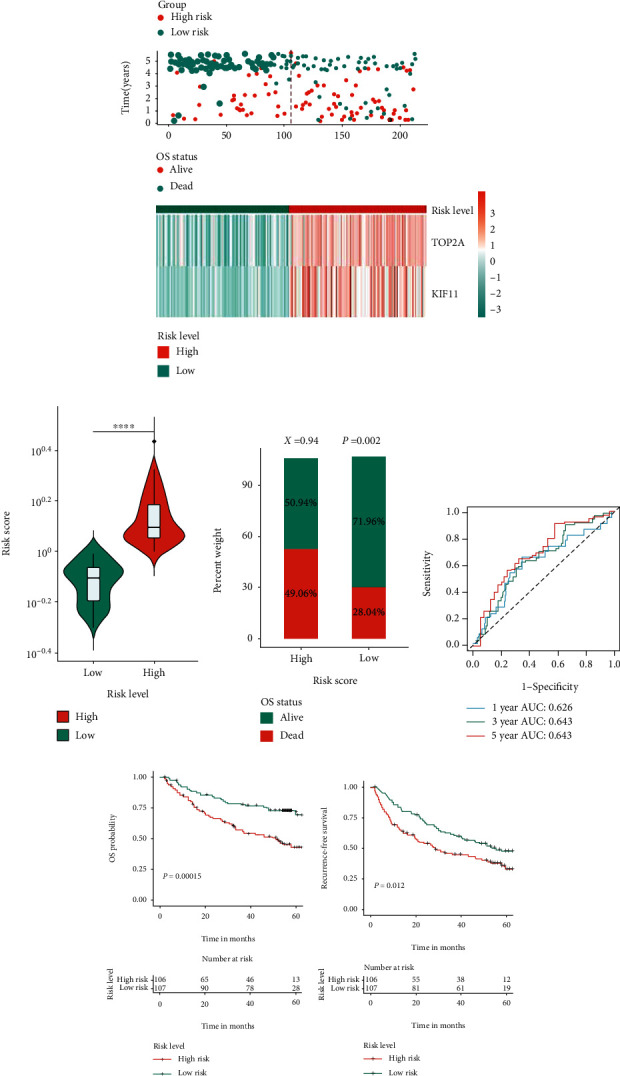
Prognostic assessments of hub genes by GSE14520 cohort. (a) Risk score, survival outcome, and hub gene expression values for each patient in high- and low-risk groups. (b) Comparison of risk scores between high- and low-risk groups. ^∗∗∗∗^*P* < 0.0001. (c) Distribute of different survival status in high- and low-risk score groups. Statistical significance was determined by chi-square test. (d) The time-dependent ROC curves for the two-hub gene-based signature at 1, 3, and 5 years to assess accuracy of prognostic prediction. (e, f) Kaplan-Meier curves of (e) OS and (f) RFS in HBV-HCC patients based on the risk score classification. *P* was calculated by the log-rank test, and *P* < 0.05 was considered statistically significant. HBV-HCC: HBV-related HCC; OS: overall survival; RFS: relapse-free survival.

To elucidate the prognostic values of the hub genes, we performed the OS survival analysis with the cohort from GSE14520, including 213 HBV-HCC patients. The resample-based log-rank test resulted in two robust prognostic hub genes (with high repentance frequency of >0.75 showing significant during resampling): TOP2A and KIF11. Then, a prognostic signature was built with these two hub genes, and all patients were assigned to high- or low-risk group based on the median risk score (0.997189). The result of risk score for each patient was shown in Figure 9(a).  Figure 9(b) suggested the significant difference of risk scores between high- or low-risk group, and OS survival rate was significant higher in low-risk group by chi-square analysis (Figure 9(c)). As for prediction accuracy, we plotted the time-dependent roc curves by risk score, and the risk signature showed the AUC values at 1, 3, and 5 years was 0.626, 0.643, and 0.693, respectively (Figure 9(d)). For Kaplan-Meier survival analyses, both the OS survival rate and the recurrence-free survival rate were shown to be significantly higher in low-risk group than high-risk group (Figures 9(e) and 9(f)).

Furthermore, we stratified patients into different risk subgroups by several clinicopathologic parameters (age, gender, ALT, AFP, level, main tumor size, cirrhosis, BCLC stage, TNM stage, and CLIP stage) using the two-hub gene-based signature. Interestingly, results showed that our classifier was still statistically significant in most subgroups (Supplementary Figure 6), suggesting its good potential and possible application to add prognostic value to the existing staging systems.

Moreover, risk levels based on the two-hub gene-based signature also suggested to be significantly associated with other aggressive clinicopathological parameters, such as TNM stage (*P* = 0.002), BCLC stage (*P* = 0.013), CLIP stage (*P* = 0.003), and alpha fetal protein (AFP) level (*P* = 0.003) by chi-square test (Table 2). Multivariate Cox regression analysis indicated that after adjusting for main tumor size, cirrhosis, TNM stage, BCLC stage, CLIP stage, and AFP level, the risk signature was still significantly correlated with OS survival outcome (HR = 1.807, 95%CI = 1.126–2.899, and *P* = 0.014), implying that the two-hub gene signature served as an independent prognostic factor for HBV-HCC patients (Table 3).

### 3.7. Gene Set Enrichment Analysis

**Figure 10:**
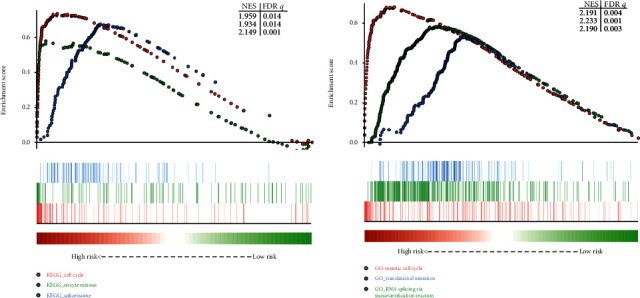
GSEA results of high- or low-risk groups divided by the two-hub gene-based signature in GSE14520 cohort. (a) Results of GO terms enriched in highly risk group vs. low-risk group. (b) Results of KEGG pathways enriched in highly risk group vs. low-risk group. GSEA: Gene Set Enrichment Analyses.

GSEA is a powerful statistical approach to identify classes of genes that are significantly associated with different disease phenotypes. Thus, inspired by the results of the prognostic analysis, we operated the GSEA to investigate the molecular mechanisms between high- and low-risk groups divided by the risk signature. GSEA was performed by KEGG at first, and top significant pathways were identified as KEGG_spliceosome (*P* < 0.001 and NES = 2.149), KEGG_cell_cycle (*P* < 0.001 and NES = 1.959), and KEGG_oocyte_meiosis (*P* < 0.001 and NES = 1.934) (Figure 10(a)). For the gene set distribution from molecular function component of the GO database, GO_translational_initiation (*P* < 0.001 and NES = 2.233), GO_meiotic_cell_cycle (*P* < 0.001 and NES = 2.191), and GO_RNA_splicing_via_transesterification_reactions (*P* < 0.001 and NES = 2.149) were ranked as the most significant terms (Figure 10(b), Supplementary Tables 2–3). These results suggested that this risk signature may exert a poorer survival for HBV-HCC patient via known crucial cancer pathways.

### 3.8. Prediction of miRNA-mRNA Interaction Network

MiRNAs have been extensively documented to regulate tumorigenesis at transcriptome level or posttranscriptional level in various cancers. Thus, we predicted the candidate miRNAs that may target these hub genes in human liver by using the miRNet online platform. Then, the miRNA-hub gene interaction network was established by Cytoscape (Supplementary Figure 7). There were 55 nodes and 77 edges involved in the network, including 16 hub genes (NDC80 was excluded) and 39 miRNAs. In the network, PRIM2, CCNA2, and RRM2 were recognized as the top 3 hub genes that had most neighbors of miRNAs, while hsa-mir-34a-5p, hsa-mir-192-5p, and hsa-mir-24-3p were the top 3 miRNAs with most targeted hub genes. The miRNA-hub gene network based on their regulatory relationships may provide a forceful basis for the further exploration of the molecular mechanisms of HBV-HCC.

## 4. Discussion

Despite the great advances in clinical management and remarkable progress in understanding the pathogenesis of HCC, the incidence and mortality rates of this malignant cancer remain unacceptably high. Chronic hepatitis B is the primary etiological factor for HCC in China and other parts of Asia [4]. With the identification of diagnostic and prognostic biomarkers of HBV-HCC, we have attempted to provide valuable insight into the molecular mechanism of HBV-HCC during tumorigenesis and development.

Public databases like GEO, TCGA, and ICGC that deposit massive datasets of high-throughput technologies like microarray and NGS platforms have facilitated the strategies for mining of integrated data, which could overcome the limitations of the small sample size in one individual cohort and heterogeneity among different studies. In the present study, we conducted DEG screening based on the transcription profiling data of GSE84402, GSE25097, GSE94660, and GSE121248 at first, and there were 309 overlapped DEGs were identified between the tumor and normal patients, comprising 100 upregulated genes and 209 downregulated genes. The result of GO analysis revealed that the upregulated DEGs were significantly enriched in the regulation of cell division activities (such as mitotic nuclear division, chromosome segregation, and nuclear division), while the downregulated DEGs were closely related to multiple cellular “catabolic process” and “biosynthetic processes” (such as organic acid catabolic process and carboxylic acid biosynthetic process). KEGG pathway analysis revealed that the upregulated DEGs were mainly involved in the cell cycle, DNA replication, oocyte meiosis, and others. Meanwhile, the downregulated DEGs were relevant to chemical carcinogenesis, retinol metabolism, tryptophan metabolism, bile secretion, and so forth.

After that, we utilized a combined strategy to identify the 17 hub genes (MCM6, MCM3, UBE2C, RRM2, RFC4, TOP2A, MCM2, CDK1, PRIM1, CCNB1, HMMR, CDC20, CCNA2, NEK2, NDC80, DLGAP5, and KIF11) from the PPI network established by the STRING database. Most of these hub genes were previously reported as oncogenes, therapeutic targets, or potential biomarkers in HCC [35–51]. We then validate the dysregulated mRNA expression levels of these hub genes using GSE14520, TCGA-LIHC, and ICGC-LIRI-JP datasets. The positive coexpression relationships of the hub genes were proved by Pearson's correlation analysis, implying the highly active interactions during the tumorigenesis. Boxplots were used to demonstrate the relevance between gene expression levels and pathological stages; however, most of the hub genes showed no significant association between early stages and late stages, stimulating us to propose that they may be used in early diagnosis for HBV-HCC. As the consequence, the ROC curves of all the 17 hub genes showed high diagnostic values for TNM stage I patients and adjacent normal tissues, suggesting their good potential in further exploiting early diagnosis, including related miRNAs, circRNAs, and aberrantly methylation markers that based on these hub genes.

For the overall survival analysis, with the aid of cohort from GSE14520, we established a risk signature with two prognostic hub genes: TOP2A and KIF11, which was demonstrated to be an independent prognostic predictor for HBV-HCC patient by univariate and multivariate analyses and was significantly correlated with tumor staging systems and AFP levels. Our GSEA result also revealed key molecular functions and KEGG pathways (especially for gene sets related to cell cycle) that involved in carcinogenesis that may be associated with OS survival stratification by the risk signature for HBV-HCC patients.

TOP2A, encoded by TOP2A gene, is a DNA topoisomerase that participates in many processes during transcription and replication through altering DNA topological structure. Previous studies confirmed that the aberrant TOP2A expression was observed in various cancer subtypes, such as breast [52], colon [53], ovarian [54], gastric [55], prostate cancer [56], and HCC [40]. In the current study, TOP2A showed high expression value in HBV-HCC, which agreed with previous results. Recent studies substantiated its oncogenic role during the tumorigenesis and development of many malignancies [52, 53]. For example, Zhang et al. found that knockdown of TOP2A could induce apoptosis and suppress cell proliferation and invasion via Akt and ERK signaling pathways in colon cancer [53]. These findings strongly imply that TOP2A may be served as an anticancer therapeutic target for clinical treatment. Actually, several TOP2A inhibitors have been approved by the US Food and Drug Administration [57], and other compounds were tested in multiple trials [58, 59]. A study on adrenocortical carcinoma (ACC) manifested that aclarubicin was the best agent of 14 TOP2A inhibitors that can decrease proliferation and tumor spheroid size in locally advanced and metastatic ACC [58]. But candidate TOP2A inhibitors with high efficacy for HCC were still rare. Considerable effort was required to explore effective reagents for HCC. Furthermore, early discoveries revealed that the elevated TOP2A expression implicated the worse overall survival for multiple cancers [52, 54, 56]. In accordance with these findings, our present study demonstrated that upregulation of TOP2A was closely related to the poor outcome for HBV-HCC patients.

KIF11 or as BimC, Eg5, belonging to kinesin superfamily, which function as nanomotors to mediate various kinds of spindle dynamics, is well known to play an essential role during cell mitosis, including chromosome positioning, bipolar spindle formation and maintenance, and antiparallel microtubule sliding, as well as microtubule crosslinking [60–62]. It may also increase translational efficiency by mediating the association of ribosomes and microtubules [63]. Recently, KIF11 has been reported to be a novel potential candidate prognostic biomarker or therapeutic target in human cancers including breast cancer [64], ovarian cancer [65], oral cancer [66], peripheral nerve sheath tumors [67, 68], and lung cancer [69]. In line with previous findings, we identified KIF11 as an oncogene during tumorigenesis of HBV-HCC, and its expression level was significantly higher in tumor samples compared with adjacent normal tissues. It was also proved to hold good potential for early detection of HBV-HCC. For prognostic analysis, KIF11 and TOP2A performed jointly well in predicting prognosis by multivariate regression. In fact, a growing body of well-known KIF11 inhibitors such as monastrol [70], S-trityl-L-cysteine (STLC) [71], HR22C16 [72], and CK0106023 [73] have been extensively studied, and small-molecule inhibitors such as Ispinesib (SB-715992) [74], Filanesib (ARRY-520) [75], and litronesib (LY2523355) [76] have entered clinical trials. However, although these inhibitors have demonstrated excellent efficacies in certain human cancer with no neurotoxicity [75, 77], none have been used as a marketed anticancer agent; thus, further investigation is warranted to in the development of KIF11-based anticancer drugs.

There are several limitations to our study. First, a larger cohort is required to further validate these results. Second, because of the cautious approach adopted in the study, we failed to enroll the adequate number of HBV-HCC cases with complete clinical characteristics and sufficient long-term follow-up, which did not allow us to conduct informative analyses of better risk stratification and validation. Third, further in-depth studies are necessary to confirm the oncogenic roles of the selected hub genes via in vitro and in vivo assays.

## 5. Conclusion

In summary, with the integrated bioinformatics analysis, 309 robust DEGs involved in HBV-HCC were screened, which is helpful for a better understanding of molecular pathogenesis and tumorigenesis of HBV-HCC. Based on a series of comprehensive downstream analysis, 17 potential hub genes were identified that may play critical roles in the development of HBV-HCC. TOP2A and KIF11 can be jointly used to predict overall survival for HBV-HCC, and all the hub genes may hold good potential in exploring early detection biomarkers and therapeutic targets for HBV-HCC.

## Figures and Tables

**Table 1:** Detailed information of selected datasets in this study.

Study	Platforms	Etiology	Tumor	Normal	Total	Technology
GSE84402	GPL570	HBV	13	13	26	Microarray
GSE25097	GPL10687	HBV	73	67	140	Microarray
GSE94660	Illumina Hiseq	HBV	21	21	42	RNA-seq
GSE121248	GPL570	HBV	70	37	107	Microarray
GSE14520	GPL3921	HBV	213	220	433	Microarray
TCGA	Illumina Hiseq	HBV	71	49	120	RNA-seq
ICGC	Illumina Hiseq	HBV	53	45	98	RNA-seq

**Table 2:** Clinicopathological features of HBV-HCC patients according to the two-hub gene-based signature in the cohort of GSE14520.

Characteristics	Low risk (*n* = 107)	High risk (*n* = 106)	*P* value
Gender			
Female	17	13	0.447
Male	90	93	
Age			
<50	48	47	0.939
≥50	59	59	
ALT level			
High (>50 U/L)	41	47	0.518
Low (≤50 U/L)	66	59	
Main tumor size^†^			
Large (>5 cm)	35	40	0.412
Small (≤5 cm)	72	65	
Cirrhosis			
Yes	96	100	0.213
No	11	6	
TNM stage			
I	56	33	0.002^∗^
II+III	51	73	
BCLC stage			
0-A	90	74	0.013^∗^
B-C	17	32	
CLIP stage			
0	58	36	0.003^∗^
≥1	49	70	
AFP level^†^			
High (≥300 ng/ml)	37	58	0.007^∗^
Low (<300 ng/ml)	67	49	

^**†**^The information was missing for certain patients. ^∗^*P* < 0.05 by *χ*^2^ test. HBV-HCC: HBV-related HCC; ALT: alanine aminotransferase; AFP: alpha fetoprotein; TNM: tumor-node-metastasis; BCLC: Barcelona Clinic Liver Cancer; CLIP: Cancer of the Liver Italian Program.

**Table 3:** Univariate and multivariate analyses of the cohort GSE14520.

Variables	Group	Univariate analysis			Multivariate analysis		
		HR	95% CI	*P* value	HR	95% CI	*P* value
Risk level	High/low	2.293	1.461-3.600	<0.001^†^	1.807	1.126-2.899	0.014^∗^
Gender	Male/female	0.599	0.289-1.242	0.168	-	-	-
Age	≥50/<50	1.190	0.771-1.837	0.432	-	-	-
ALT level (U/L)	≥50/<50	1.107	0.716-1.712	0.648	-	-	-
Main tumor size (cm)	≥5/<5	2.063	1.330-3.199	0.001^†^	1.269	0.77-2.091	0.349
Cirrhosis	Yes/no	0.224	0.055-0.910	0.036^†^	0.349	0.084-1.440	0.145
TNM stage	II+III/I	0.348	0.210-0.577	<0.001^†^	0.579	0.326-1.027	0.061
BCLC stage	B-C/0-A	0.283	0.18-0.445	<0.001^†^	0.478	0.27-0.848	0.012^∗^
CLIP stage	≥1/0	2.133	1.338-3.398	0.001^†^	1.129	0.549-2.322	0.741
AFP level (ng/ml)	≥300/<300	1.572	1.019-2.426	0.041^†^	1.006	0.541-1.869	0.986

^†^Significant in univariate Cox regression and were further enrolled for multivariable Cox regression analysis. ^∗^*P* < 0.05 by both univariate and multivariate analyses. AFP: alpha fetoprotein; TNM: tumor-node-metastasis; ALT: alanine aminotransferase; BCLC: Barcelona Clinic Liver Cancer; CLIP: Cancer of the Liver Italian Program.

## Data Availability

The data used to support our results are available at the GEO (https://www.ncbi.nlm.nih.gov/geo/), TCGA (https://portal.gdc.cancer.gov/), and ICGC (https://icgc.org/).

## Supplementary Materials

Supplementary Materials “Supplementary Figure 1: flow chart of the prognostic key gene identification with “multi-split” method. Supplementary Figure 2: validation of 309 overlapping DEGs by integrating analysis with batch removal. Supplementary Figure 3: PPI network of DEGs. Supplementary Figure 4: boxplots showing the relative expression levels of 17 hub genes across normal liver tissues and cancer tissues with different BCLC stages for HBV-HCC. Supplementary Figure 5: the ROC curves of the selected hub genes to evaluate the diagnostic efficiency for HBV-HCC based on TCGA-LIHC cohort (A-C) and GSE14520 (D-F) cohort. Supplementary Figure 6: stratification analysis of the two-hub gene-based classifier. Supplementary Figure 7: miRNA-mRNA interaction network predicted by miRNet.”. Supplementary Table 1: validation of 309 differentially expressed genes (DEGs) by integrating analysis. Supplementary Table 2: Gene Set Enrichment Analysis (GSEA) result of the two-hub gene-based prognostic signature by the KEGG database. Supplementary Table 3: Gene Set Enrichment Analysis (GSEA) result of the two-hub gene-based prognostic signature by molecular function component of the GO database.
