# Supplementary material for: Identification of Potential Hub Genes Related to Diagnosis and Prognosis of Hepatitis B Virus-Related Hepatocellular Carcinoma via Integrated Bioinformatics Analysis
Source: Biomed Res Int. 2020 Dec 8;2020:4251761. doi: 10.1155/2020/4251761 (PMC7744201; doi:10.1155/2020/4251761)

**Supplementary Figure 1 Flow chart of the prognostic key genes identification with “multi-split” method.**

**Supplementary Figure 2 Validation of 309 overlapping DEGs by integrating analysis with batch removal.** (a) Principal components analysis (PCA) plot for all the samples from GSE25097, GSE84402, and GSE121248. (b) Heatmap of 309 DEGs showing the expression value in each patient. Each row represents a sample, and each column represents a gene. Red indicates high expression level, while green indicates low expression level. DEGs, differentially expressed genes.

**Supplementary Figure 3 PPI network of DEGs**

Totally 209 DEGs were selected by the STRING online tool to construct the PPI network. Red nodes denote the 84 upregulated DEGs, and green nodes denote the 125 downregulated DEGs, while the edge between two nodes denote the interaction of two proteins. DEGs, differentially expressed genes.

**Supplementary Figure 4 boxplots showing the relative expression levels of 17 hub genes across normal liver tissues and cancer tissues with different BCLC stages for HBV-HCC**. *p < 0.05, **p < 0.01, ***p < 0.001, ****p < 0.0001. HBV-HCC, HBV-related HCC.

**Supplementary Figure 5 The ROC curves of the selected hub genes to evaluate the diagnostic efficiency for HBV-HCC based on TCGA-LIHC cohort (A-C) and GSE14520(D-F) cohort.** colored lines denote sensitive curves for each hub gene, and grey line denotes the identify line. ROC, Receiver operating characteristic; AUC, area under the curve. HBV-HCC, HBV-related HCC.

**Supplementary Figure 6 Strati­fication analysis of the two-hub genes-based classifier**. Kaplan-Meier survival analysis to evaluate the prognostic effect of the two-hub genes-based classifier in subgroups of age, gender, ALT level, AFP level, main tumor size, cirrhosis, BCLC stage, TNM stage, and CLIP stage.

**Supplementary Figure 7 miRNA-mRNA interaction network predicted by miRNet.** Diamond nodes indicate the hub-mRNAs and rectangle nodes indicate the miRNAs that may target these genes.

**Supplementary Figure 1**


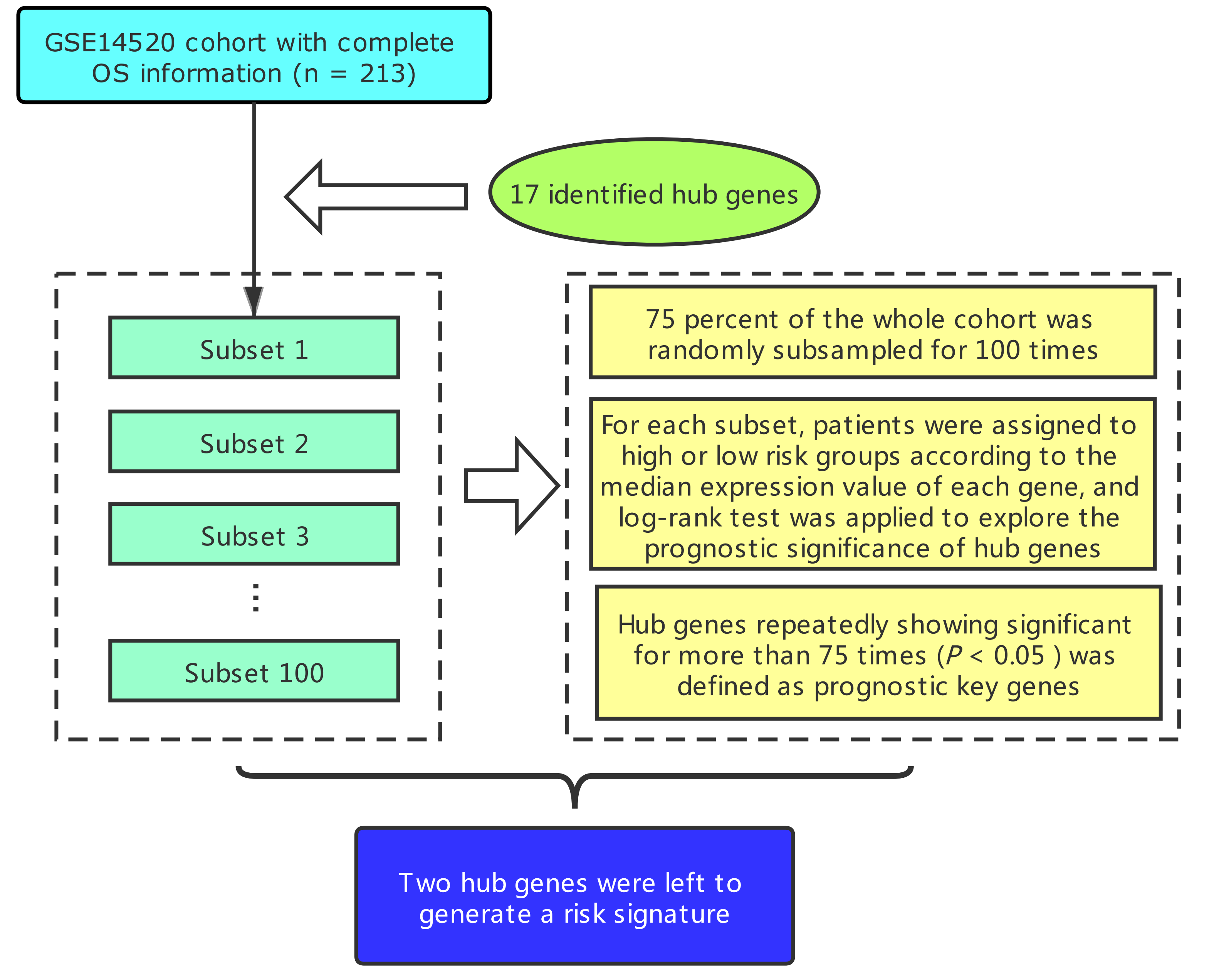


**Supplementary Figure 2**


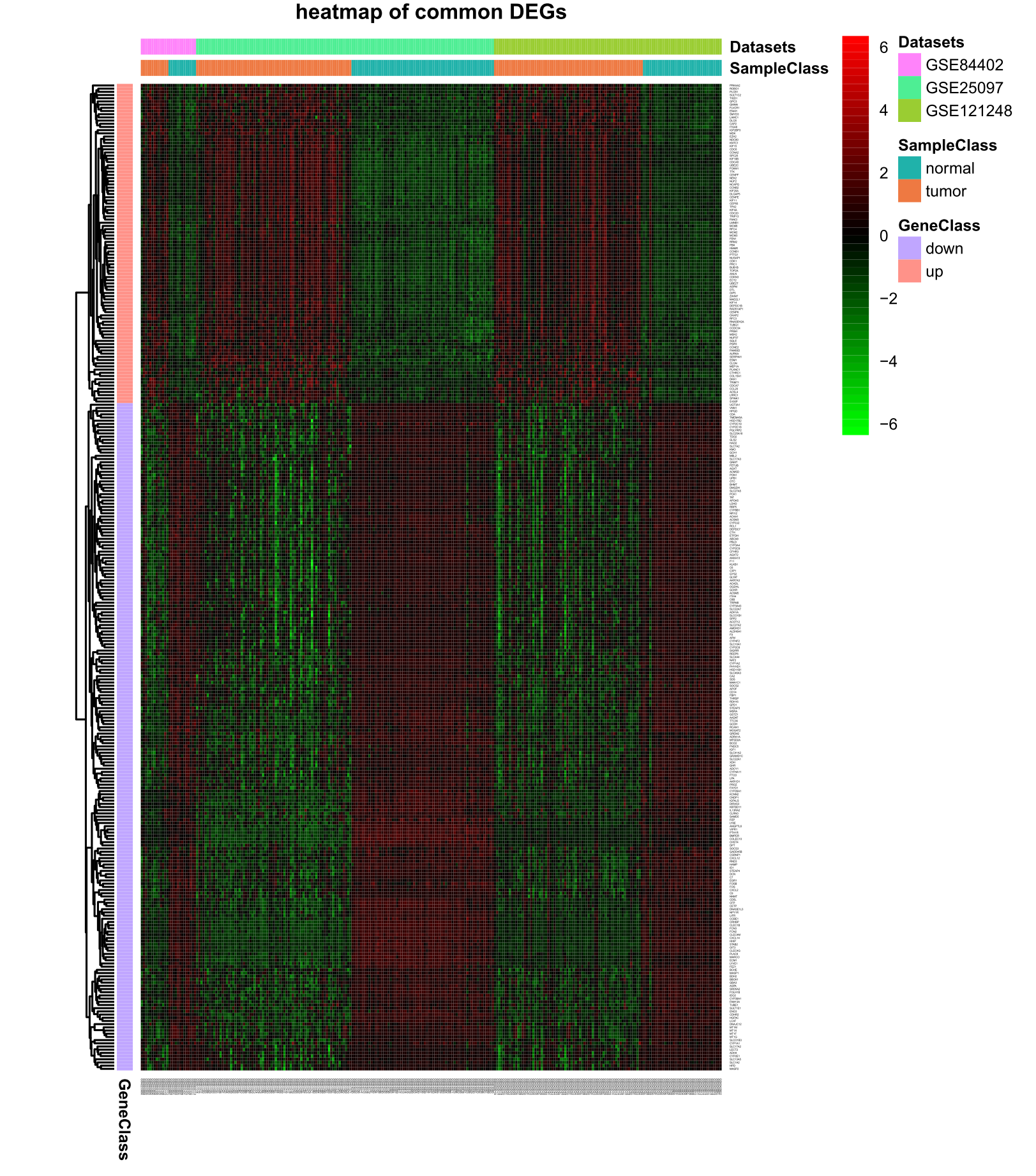

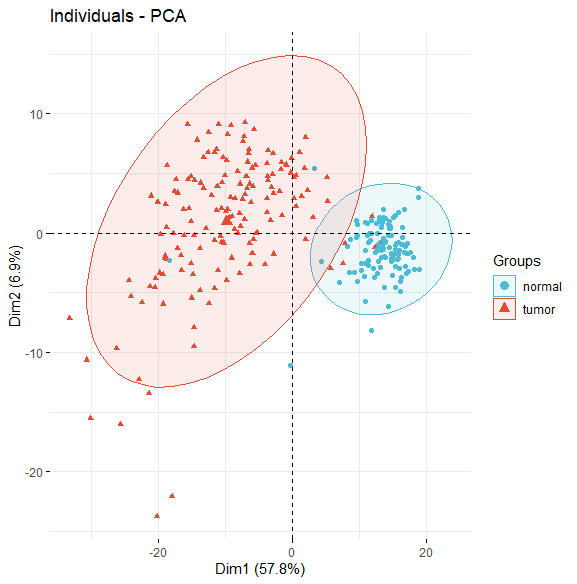


**a**

**b**

**Supplementary Figure 3**

**
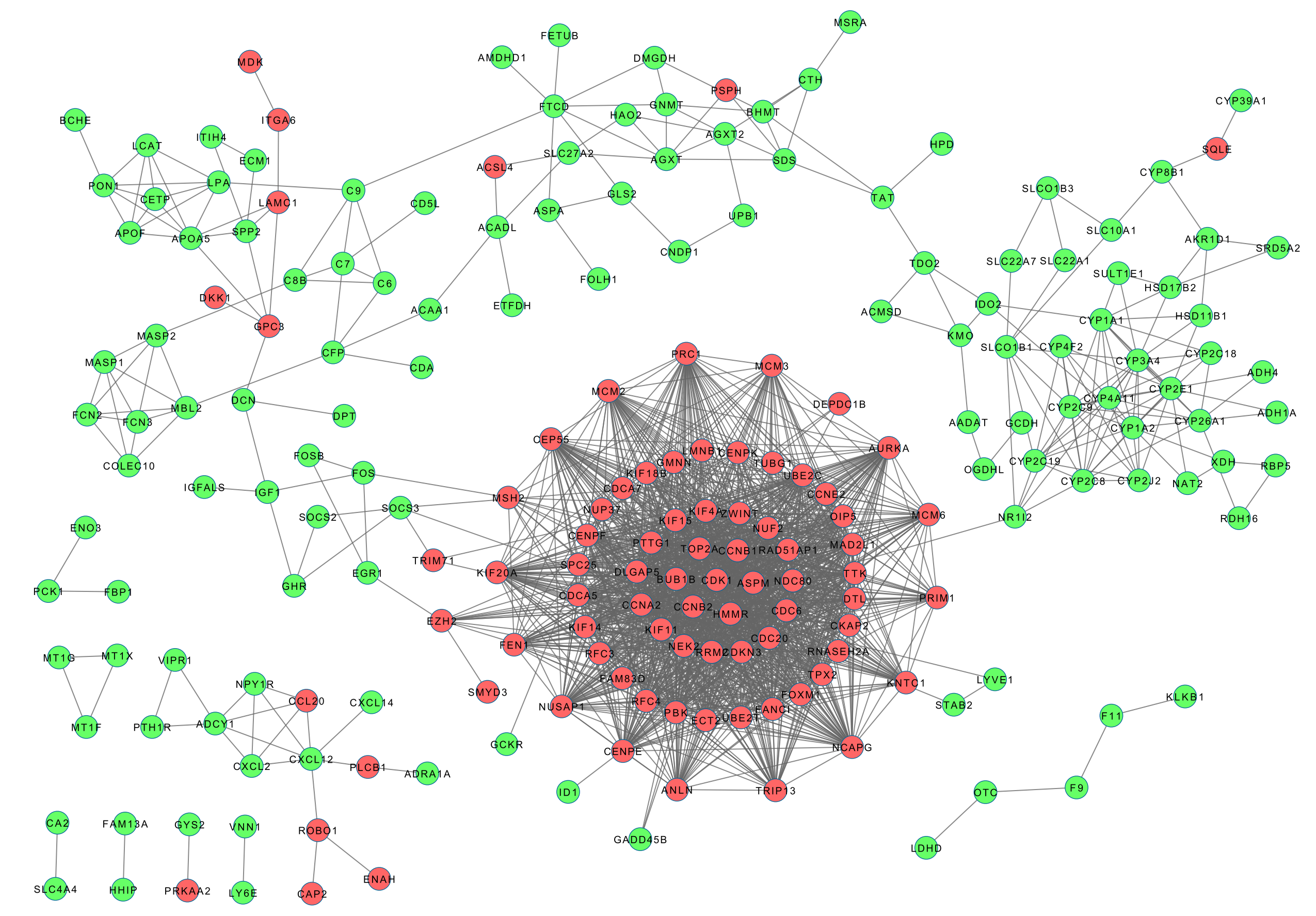
**

**Supplementary Figure 4**


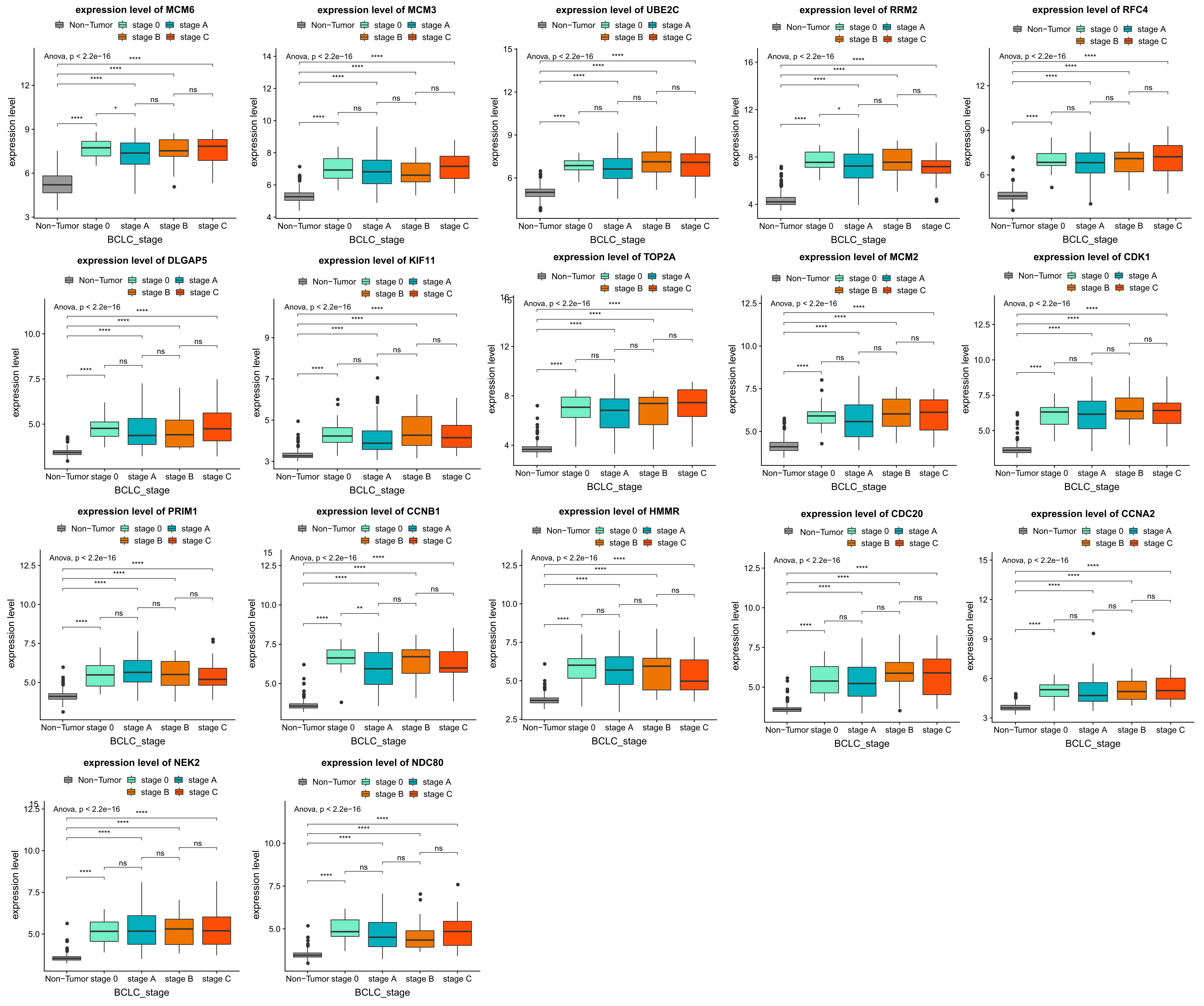


**Supplementary Figure 5**


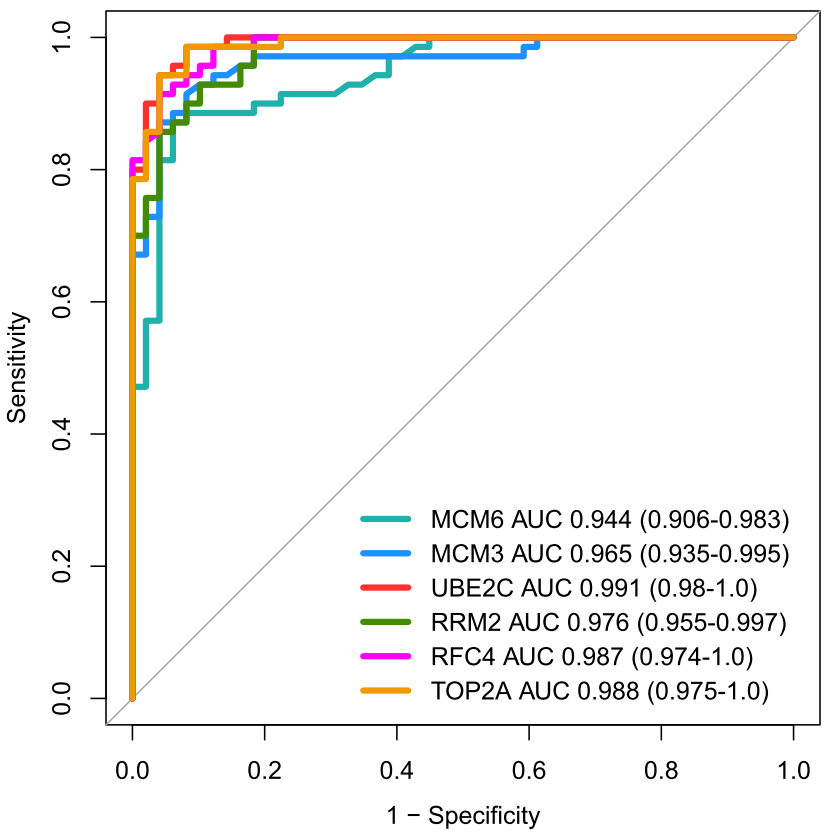

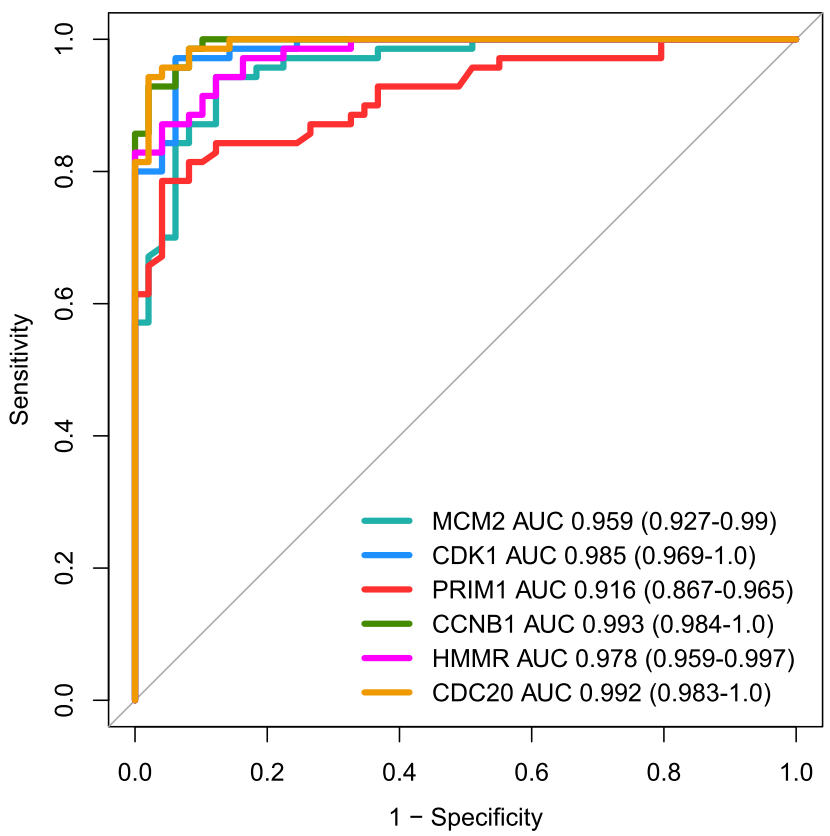

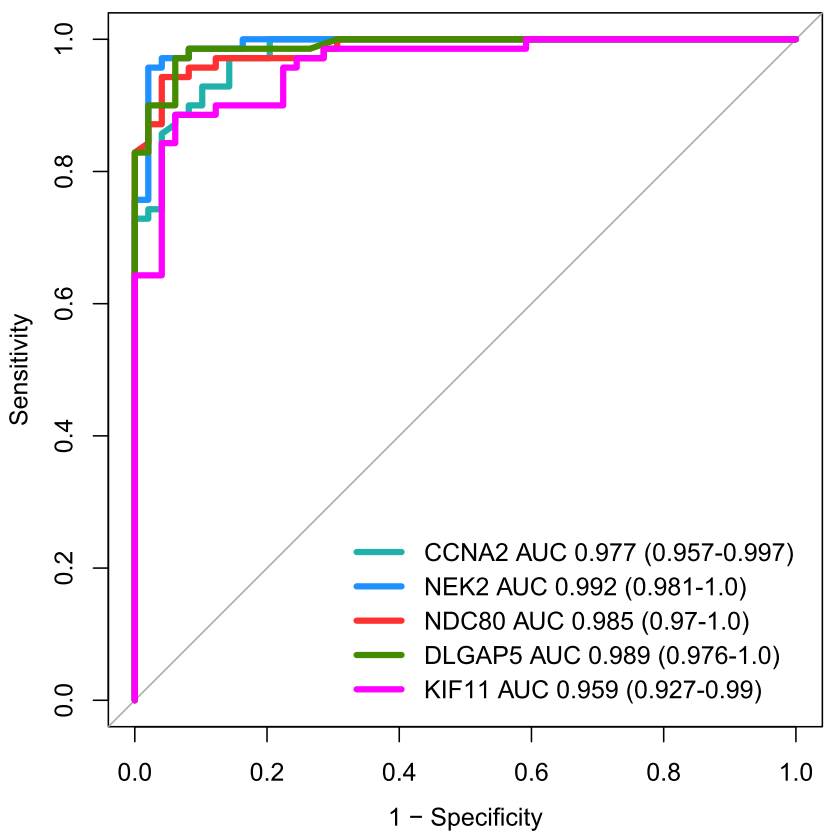

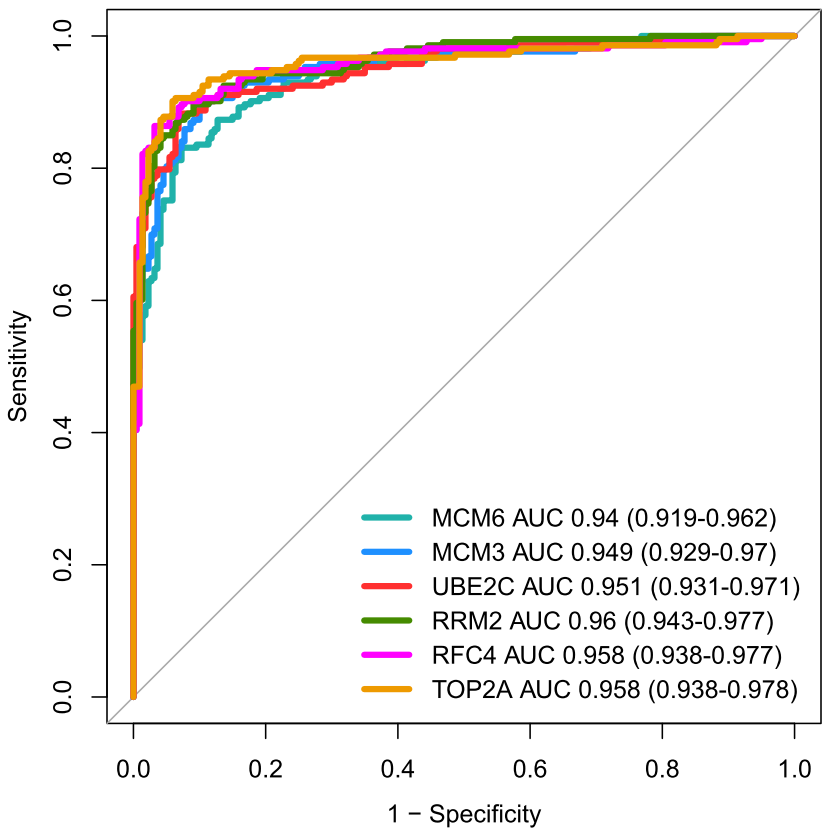

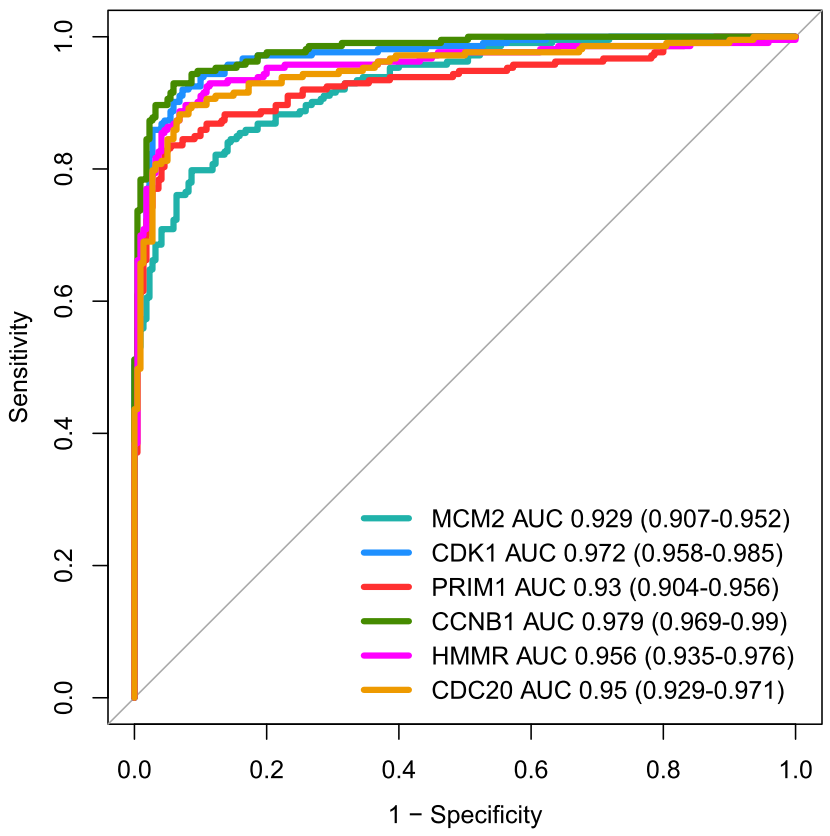

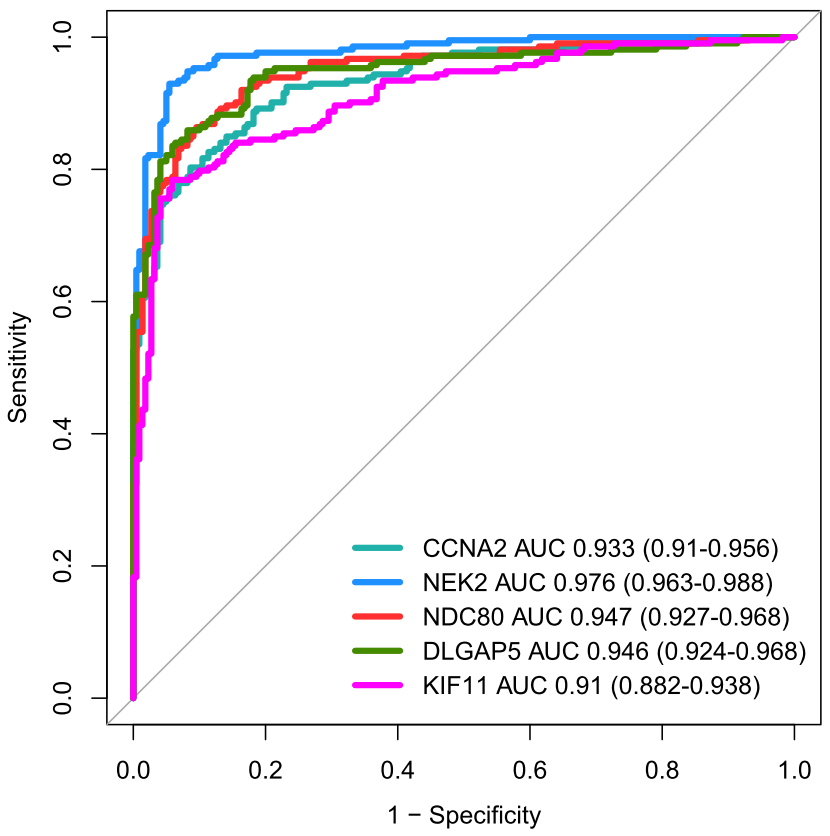


**a**

**d**

**b**

**c**

**e**

**f**

**Supplementary Figure 6**


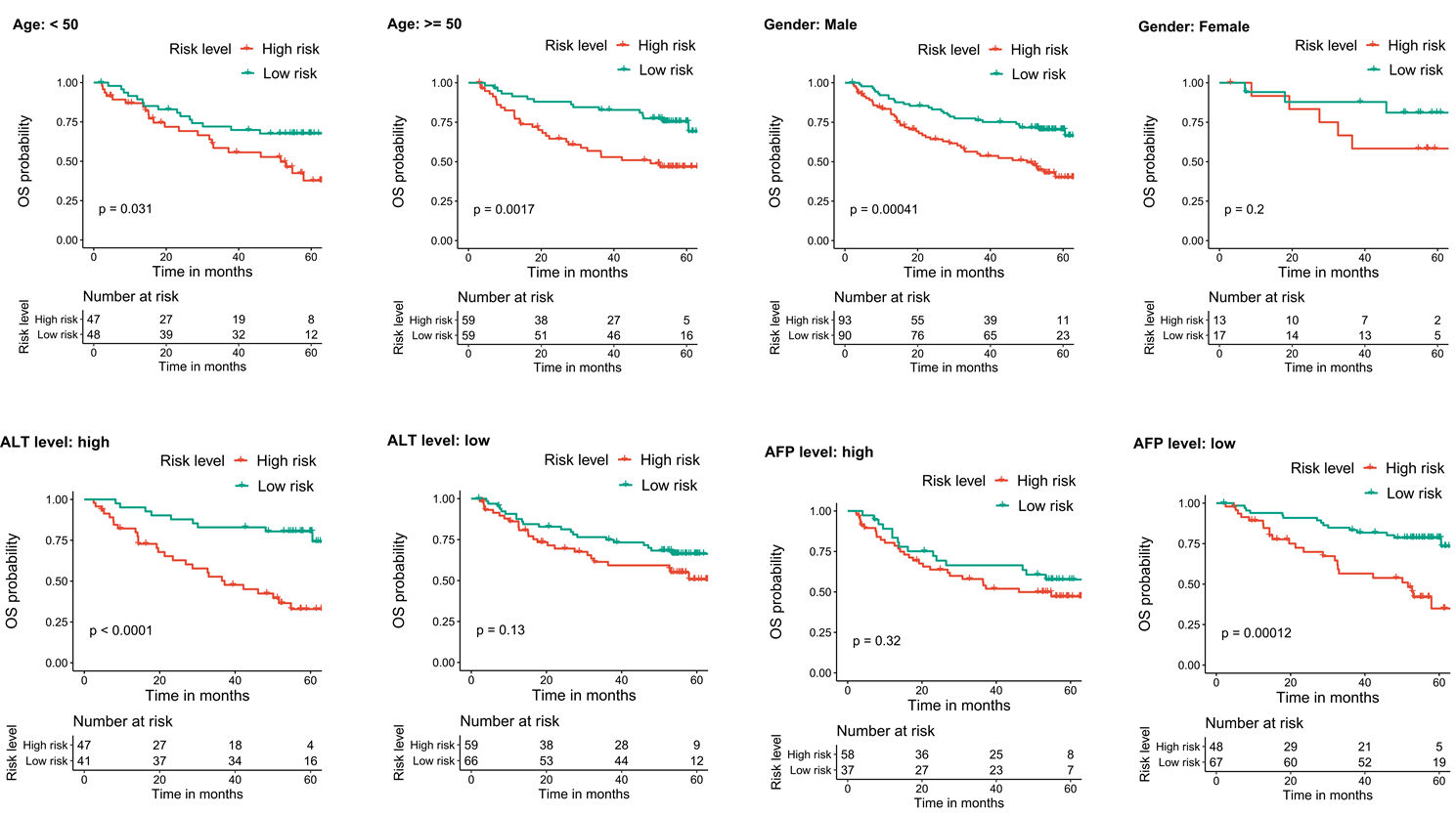

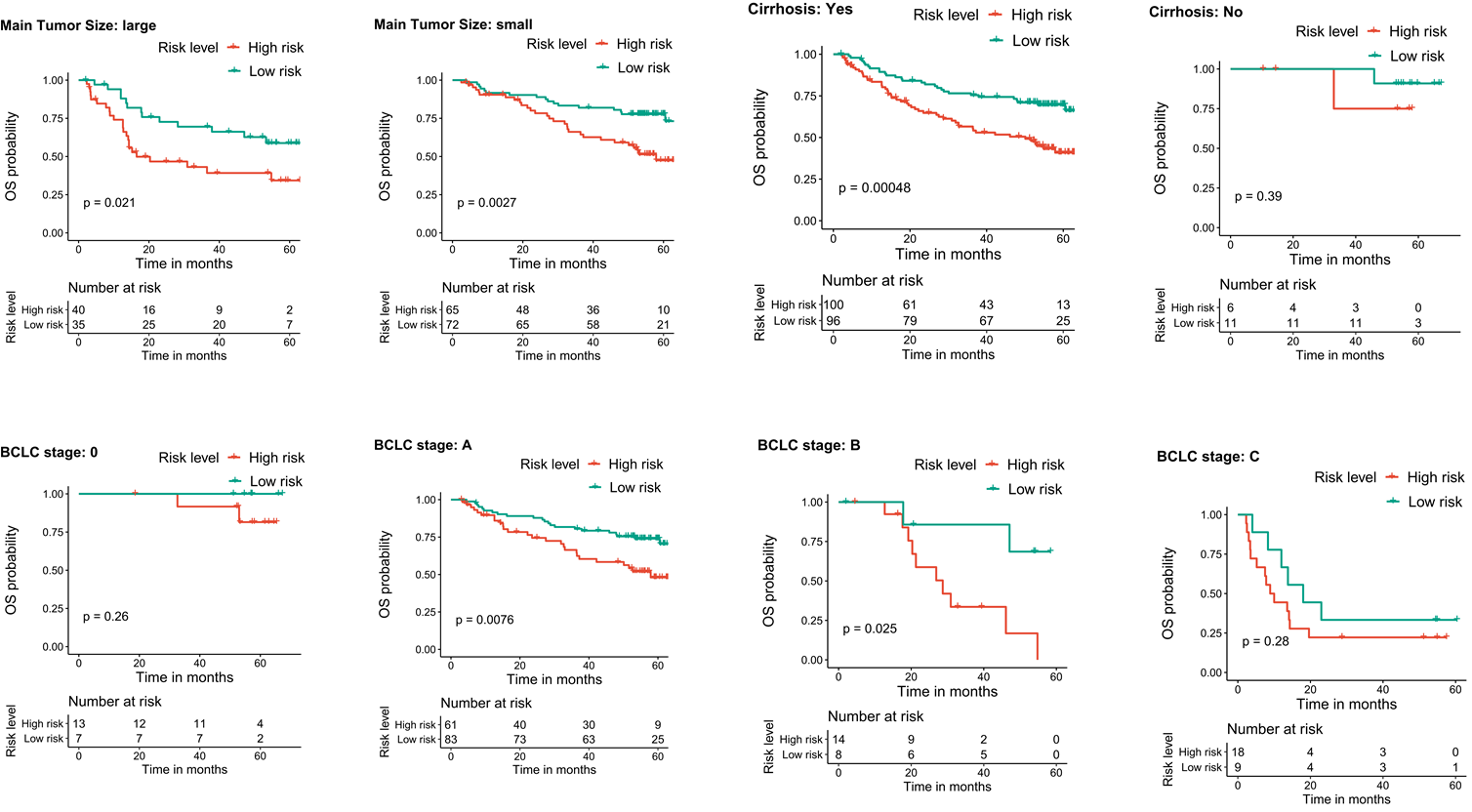

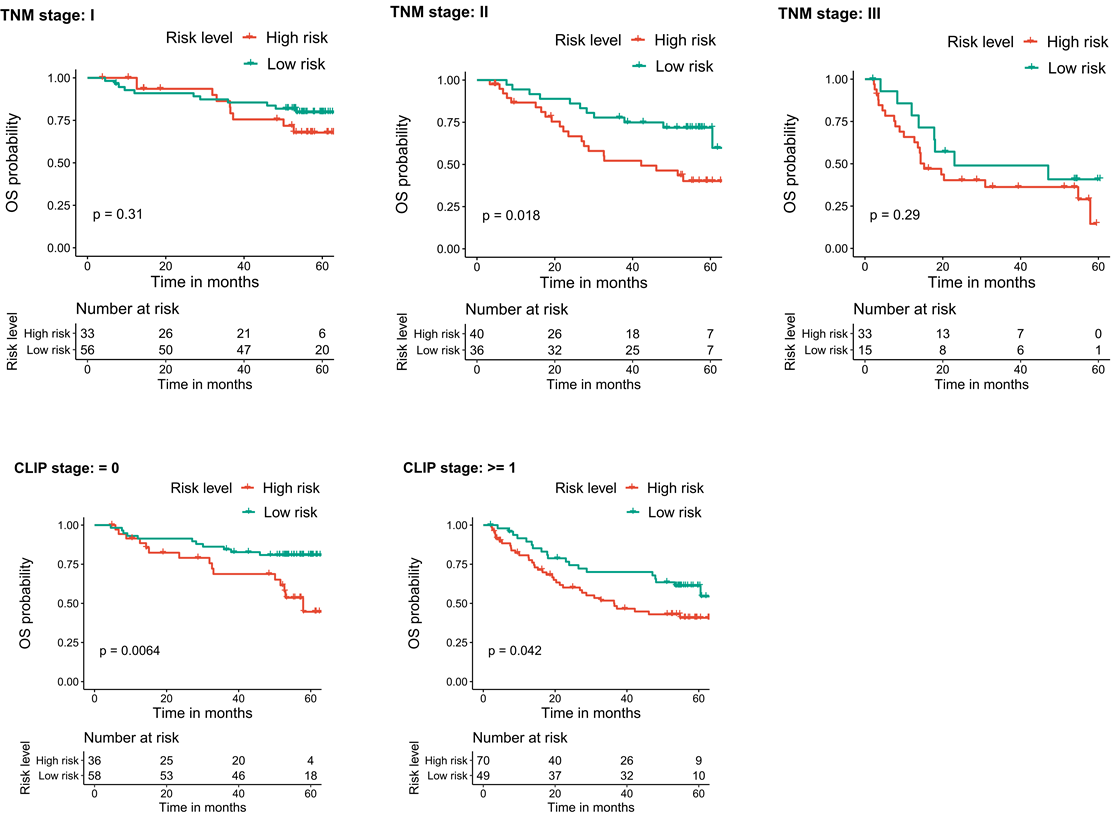


**Supplementary Figure 7**


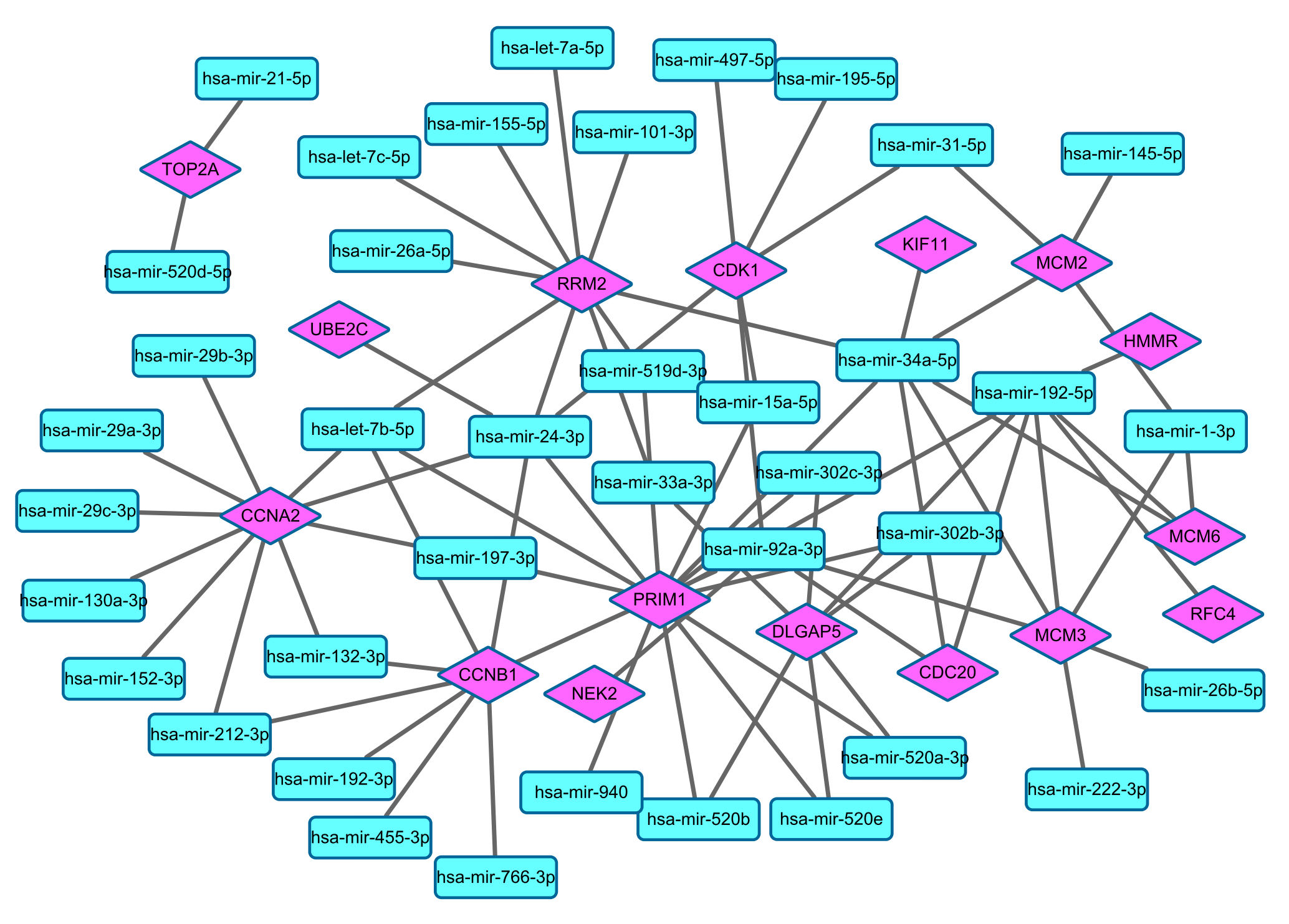

Supplement: Supplementary Materials — “Supplementary Figure 1: flow chart of the prognostic key gene identification with “multi-split” method. Supplementary Figure 2: validation of 309 overlapping DEGs by integrating analysis with batch removal. Supplementary Figure 3: PPI network of DEGs. Supplementary Figure 4: boxplots showing the relative expression levels of 17 hub genes across normal liver tissues and cancer tissues with different BCLC stages for HBV-HCC. Supplementary Figure 5: the ROC curves of the selected hub genes to evaluate the diagnostic efficiency for HBV-HCC based on TCGA-LIHC cohort (A-C) and GSE14520 (D-F) cohort. Supplementary Figure 6: stratification analysis of the two-hub gene-based classifier. Supplementary Figure 7: miRNA-mRNA interaction network predicted by miRNet.”. Supplementary Table 1: validation of 309 differentially expressed genes (DEGs) by integrating analysis. Supplementary Table 2: Gene Set Enrichment Analysis (GSEA) result of the two-hub gene-based prognostic signature by the KEGG database. Supplementary Table 3: Gene Set Enrichment Analysis (GSEA) result of the two-hub gene-based prognostic signature by molecular function component of the GO database. [file 4251761.f1.zip › mat.4251761.v3.docx]
